# Protein Data Bank: A Comprehensive Review of 3D Structure Holdings and Worldwide Utilization by Researchers, Educators, and Students

**DOI:** 10.3390/biom12101425

**Published:** 2022-10-04

**Authors:** Stephen K. Burley, Helen M. Berman, Jose M. Duarte, Zukang Feng, Justin W. Flatt, Brian P. Hudson, Robert Lowe, Ezra Peisach, Dennis W. Piehl, Yana Rose, Andrej Sali, Monica Sekharan, Chenghua Shao, Brinda Vallat, Maria Voigt, John D. Westbrook, Jasmine Y. Young, Christine Zardecki

**Affiliations:** 1 Research Collaboratory for Structural Bioinformatics Protein Data Bank, Rutgers, The State University of New Jersey, Piscataway, NJ 08854, USA; 2 Institute for Quantitative Biomedicine, Rutgers, The State University of New Jersey, Piscataway, NJ 08854, USA; 3 Cancer Institute of New Jersey, Rutgers, The State University of New Jersey, New Brunswick, NJ 08901, USA; 4 Research Collaboratory for Structural Bioinformatics Protein Data Bank, San Diego Supercomputer Center, University of California San Diego, La Jolla, CA 92093, USA; 5 Department of Chemistry and Chemical Biology, Rutgers, The State University of New Jersey, Piscataway, NJ 08854, USA; 6 Research Collaboratory for Structural Bioinformatics Protein Data Bank, Department of Bioengineering and Therapeutic Sciences, Department of Pharmaceutical Chemistry, Quantitative Biosciences Institute, University of California San Francisco, San Francisco, CA 94158, USA

**Keywords:** Protein Data Bank, Open Access, Worldwide Protein Data Bank, macromolecular crystallography, cryogenic electron microscopy, cryogenic electron tomography, electron crystallography, micro-electron diffraction, nuclear magnetic resonance spectroscopy, biological macromolecules, proteins, nucleic acids, DNA, RNA, carbohydrates, small-molecule ligands

## Abstract

The Research Collaboratory for Structural Bioinformatics Protein Data Bank (RCSB PDB), funded by the United States National Science Foundation, National Institutes of Health, and Department of Energy, supports structural biologists and Protein Data Bank (PDB) data users around the world. The RCSB PDB, a founding member of the Worldwide Protein Data Bank (wwPDB) partnership, serves as the US data center for the global PDB archive housing experimentally-determined three-dimensional (3D) structure data for biological macromolecules. As the wwPDB-designated Archive Keeper, RCSB PDB is also responsible for the security of PDB data and weekly update of the archive. RCSB PDB serves tens of thousands of data depositors (using macromolecular crystallography, nuclear magnetic resonance spectroscopy, electron microscopy, and micro-electron diffraction) annually working on all permanently inhabited continents. RCSB PDB makes PDB data available from its research-focused web portal at no charge and without usage restrictions to many millions of PDB data consumers around the globe. It also provides educators, students, and the general public with an introduction to the PDB and related training materials through its outreach and education-focused web portal. This review article describes growth of the PDB, examines evolution of experimental methods for structure determination viewed through the lens of the PDB archive, and provides a detailed accounting of PDB archival holdings and their utilization by researchers, educators, and students worldwide.

## 1. Introduction

The Protein Data Bank (PDB) is now in its 51st year of continuous operations. As the first open-access digital data resource in biology, it was established in 1971 with just seven protein structures [1]. At the time of writing, PDB holdings numbered nearly 200,000 experimentally-determined three-dimensional (3D) structures of proteins and nucleic acids (DNA and RNA) and their complexes with one another and small-molecule ligands (e.g., enzyme co-factors, drugs, investigational agents). Since 2003, the PDB archive has been jointly managed by the Worldwide Protein Data Bank (wwPDB, wwpdb.org, accessed on 28 August 2022) partnership [2,3]. wwPDB Full Members include the US-funded Research Collaboratory for Structural Bioinformatics Protein Data Bank (RCSB PDB, RCSB.org, [4,5,6,7]); Protein Data Bank in Europe (PDBe, PDBe.org, [8]); Protein Data Bank Japan (PDBj, PDBj.org, accessed on 28 August 2022 [9]); the Electron Microscopy Data Bank (EMDB, emdb-empiar.org, accessed on 28 August 2022 [10,11]); and the Biological Magnetic Resonance Bank (BMRB, bmrb.io, accessed on 28 August 2022 [12,13]). The activities of the wwPDB are governed by a charter, which was last renewed in 2021 on the occasion of the accession of EMDB (www.wwpdb.org/about/agreement, accessed on 28 August 2022). The RCSB PDB is headquartered at Rutgers, The State University of New Jersey with smaller teams based at the University of California San Diego (UCSD) and the University of California San Francisco (UCSF). Within the wwPDB, RCSB PDB serves as the designated Archive Keeper for the PDB, responsible for safeguarding both digital information and a physical archive of correspondence. A conservative estimate of USD 100,000 for the average replacement cost of each individual PDB structure translates to a replacement cost of the structures in the entire archive of nearly USD 20 billion (as of mid-2022).

wwPDB partners are committed to the FAIR (Findability, Accessibility, Interoperability, and Reusability [14]) and FACT (Fairness, Accuracy, Confidentiality, and Transparency [15]) Principles emblematic of responsible data stewardship in the modern era. The PDB archive has been accredited by CoreTrustSeal (coretrustseal.org accessed on 28 August 2022). Since its inception, the PDB has been regarded as a pioneer in the open-access data movement. More than 60,000 structural biologists working on every inhabited continent have generously deposited 3D structure information (atomic coordinates, experimental data, and related metadata) to the archive over more than fifty years. Today, many millions of PDB data consumers worldwide working in fundamental biology, biomedicine, bioengineering, biotechnology, and energy sciences enjoy no-cost access to 3D biostructure information with no limitations on data usage. Many scientific research areas have been profoundly impacted by the creation and availability of the PDB archive [16,17,18,19,20,21,22,23,24,25,26,27,28,29,30,31,32,33,34,35,36,37,38,39,40,41,42].

This review article is published in a Special Issue of *Biomolecules* honoring Professor Phil Bourne, who served as Associate Director of the RCSB PDB from 1998–2014. Phil led the UCSD site, where he focused on database development, integration with the scientific literature, and PDB search and data visualization tools. Bourne and Helge Weissig played critical roles in developing the inaugural version of the RCSB PDB data-delivery web portal at RCSB.org [4,43]. Access to PDB data and development of tools for query, visualization, and analysis as supported by the wwPDB partnership have helped drive the growth of structural and computational biology. PDB data and its usage by researchers, educators, and students over more than five decades is presented to highlight the evolution of these scientific fields and inform the next fifty years of successful PDB operations.

## 2. Results

### 2.1. PDB Data Metrics and Trends

**Figure 1:**
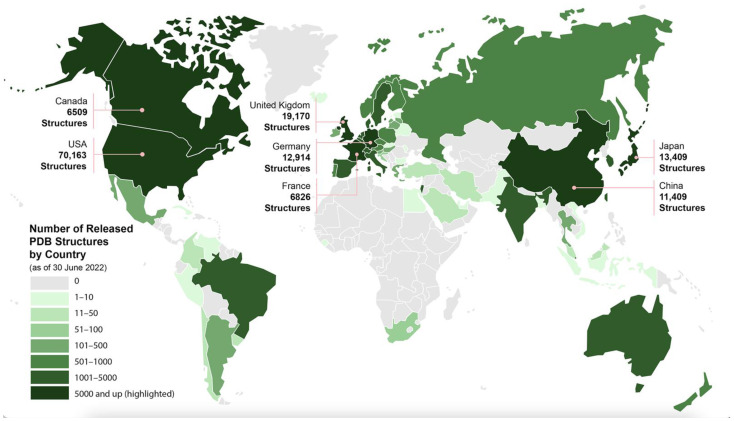
Geographic distribution of PDB depositions from 1971 to mid-2022.

Since 1971, PDB structures have been contributed freely by more than sixty thousand structural biologists (depositors) working on every permanently inhabited continent (Figure 1). Structural biologists in 53 countries, territories, etc. recognized by the United Nations deposited data to PDB during 2021. All used the wwPDB OneDep software system (deposit.wwpdb.org) that enables complete structure data deposition [44], rigorous validation [45,46], and expert biocuration [47]. OneDep currently supports 3D macromolecular structures determined using the following experimental methods: macromolecular crystallography (MX), 3D electron microscopy (3DEM), nuclear magnetic resonance (NMR) spectroscopy, electron crystallography (EC), and micro-electron diffraction (microED). Currently, newly deposited structures are processed at RCSB PDB (Americas, Oceania), PDBe (Europe, Africa), or PDBj (Asia, Middle East), allocated based on the depositor’s IP address location.

**Figure 2:**
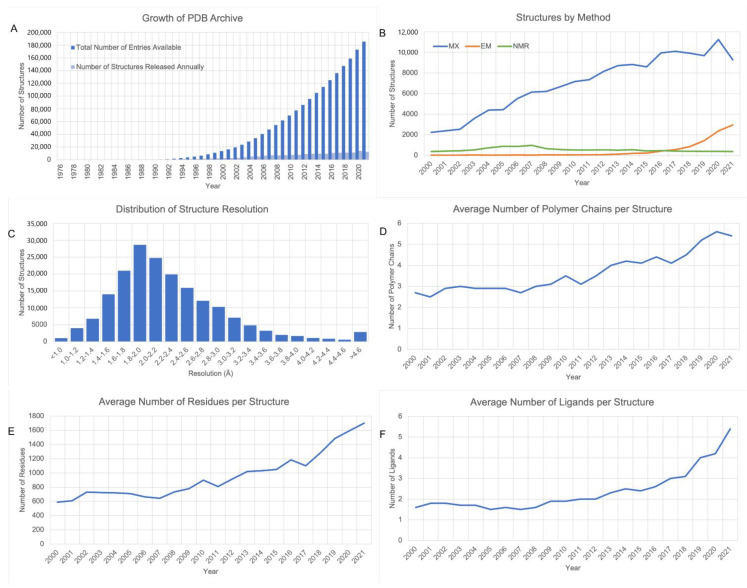
PDB archive metrics. (**A**). Growth 1976–2021. (**B**). New MX, 3DEM, and NMR structures released annually (2000–2021). (**C**). MX and 3DEM structure counts vs. resolution (Å). (**D**). Average number of residues per structure for structures released annually (2000–2021). (**E**). Average number of polymer chains per structure for structures released annually (2000–2021). (**F**). Average number of non-polymer ligands per structure for structures released annually (2000–2021).

Figure 2A illustrates growth of the PDB archive over the past 50+ years. Since the first X-ray crystal structure of a protein (sperm whale myoglobin) was determined by Sir John Kendrew and his colleagues [48], the discipline has become central to molecular and cellular biology. Figure 2B documents the impact of MX, 3DEM, and NMR on annual PDB data releases. Since 2016, annual releases of PDB MX structures have plateaued at ~10,000, with the exception of substantial spike in 2020 driven by the pandemic lockdown and various MX-based fragment screening campaigns against SARS-CoV-2 proteins thought to represent good drug discovery targets. During the same period, NMR structure releases declined, and 3DEM structure releases grew exponentially (increasing ~6-fold in only 4 years). As of mid-2022, the archive contained 166,894 MX structures, 11,294 3DEM structures, and 13,738 NMR structures. Given current deposition metrics, aggregate 3DEM structure holdings are expected to surpass those of NMR in late 2022 or early 2023. Of immediate importance to those working to combat the COVID-19 pandemic, the PDB archive currently holds >2600 SARS-CoV-2 related structures (~800 released in 2020, and ~900 released in 2021). Figure 2C shows the number of PDB MX and 3DEM structures broken down as a function of resolution (median value ~2.0 Å). While nearly all PDB structures determined at better than 2.5 Å resolution came from MX (~99.6%), 3DEM is now capable of delivering structures to nearly 1Å resolution (e.g., 1.15 Å resolution structure of apoferritin, PDB ID 7a6a [49]).

While the total number of PDB structures continues to grow, their complexity is increasing year-on-year. Figure 2D illustrates structure complexity as a function of time as judged by the average number of amino acid and/or nucleotide residues per PDB ID. As of mid-2022, the total number of residues (proteins and nucleic acid) in the archive exceeded 200 million and the total number of atoms exceeded 1.5 billion. Figure 2E,F show similar trends for the average number of polymer chains per PDB ID and average number of ligands per PDB ID (excluding bound water molecules, other solvents, salts, ions, common buffers, crystallization and cryoprotection agents as specified in Shao et al. [50]), respectively.

### 2.2. Evolution of Structural Biology Methods Viewed through the Lens of the PDB

As evidenced in Figure 2A, growth of the PDB has been much faster than linear. This section examines the evolution of structural biology as a discipline viewed through the lens of PDB archival holdings. Technical innovations in MX, 3DEM, and NMR are discussed in some detail, followed by a brief account of the emergence of microED as an exciting new diffraction method for structure determination of biological macromolecules.

### 2.3. Macromolecular Crystallography (MX)

**Figure 3:**
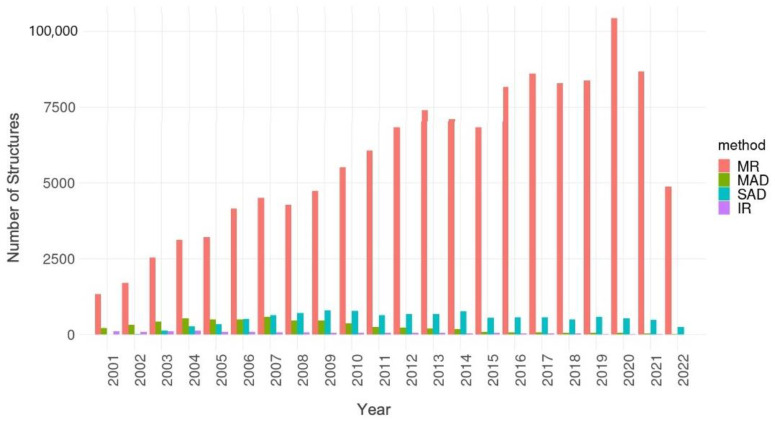
PDB MX structure phasing method trends vs. year of structure release from 2001–2021 (MR: molecular replacement; MAD: multi-wavelength anomalous dispersion; SAD: single-wavelength anomalous dispersion; IR: isomorphous replacement).

Structures determined using the MX method were the first to be deposited into the PDB. All of these early structures were determined using isomorphous replacement (IR) [51] to solve the crystallographic phase problem. Slow but steady growth of the PDB archive during the 1980s combined with development of the molecular replacement (MR) method for structure determination by Michael Rossmann [52] helped to accelerate MX. In 2001, after PDB first began systematic collection of phasing method information, it was already apparent that most 3D structures being deposited to the archive were determined using MR. Figure 3 also shows that by 2001 IR had been largely abandoned as a de novo structure determination method in favor of multiple-wavelength anomalous dispersion (MAD, to be supplanted by single-wavelength anomalous dispersion or SAD) for new structure determinations for which MR was not feasible. Analyses across the entire archive revealed that MR was used to determine ~85% of all PDB MX structures as of mid-2022. This method depends critically on the parsimony of macromolecular evolution. Protein domain folds (3D structures) are reused repeatedly within biomolecules carrying out similar biochemical or biological functions. According to generally accepted estimates, ~10,000 distinct polypeptide chain folds account for the vast majority of naturally occurring proteins.

The other important trend in MX structure determination practices evident from historical PDB data concerns X-ray sources. Widespread availability of MX beamlines at synchrotron radiation sources transformed how protein crystallographers work. As of mid-2022, ~85% of PDB MX structures relied on diffraction data collected at synchrotrons vs. ~15% that used home X-ray sources. Before 2000, most PDB MX structures released annually were the products of home sources. In contrast, only ~7% of new PDB MX structures came from home sources during the period of 2017 through 2021. Among global synchrotron sources worldwide, the top five contributors of PDB MX structures in rank order as of mid-2022 were the Advanced Photon Source (APS, ~21% of all PDB MX structures), the European Synchrotron Research Facility (ESRF, ~12%), Diamond (~9%), the Advanced Light Source (ALS, ~7%), and the National Synchrotron Light Source (NSLS, ~6%). Three of these top five biostructure-producing synchrotrons (APS, ALS, and NSLS) and others operated by the US Department of Energy contributed ~41% of all PDB MX structures worldwide as of mid-2022.

**Figure 4:**
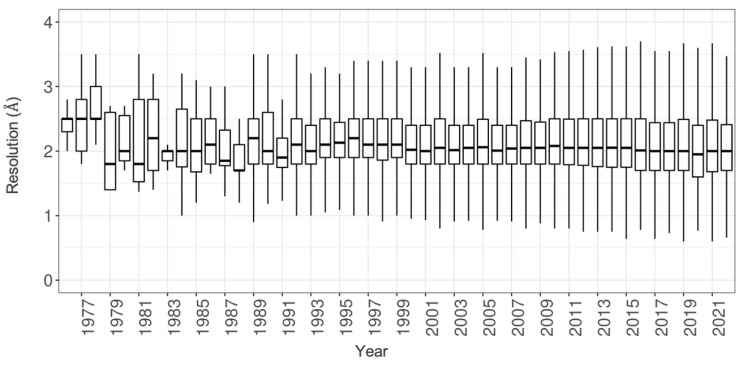
Box plot display of PDB MX structure resolution vs. time. The bold solid bar within each box corresponds to the median value for structures publicly released that year. (N.B.: Small numbers of extreme outliers with resolution > 4 Å were excluded from this analysis for clarity).

Given the critical roles played by synchrotron radiation sources in MX structure studies, one could reasonably expect that bright X-ray sources combined with cryogenic data collection would have contributed to ongoing improvements in structure resolution throughout the history of the PDB. Figure 4 tells an entirely different story. As of 1990, well before access to synchrotron beamlines and cryo-cooling of protein crystals became routine, median resolution of new MX structures released by the PDB annually plateaued at ~2.0 Å. Since then, median resolution of PDB MX structures has not changed appreciably. This reality almost certainly reflects limitations due to the degree of order (or disorder) typical of crystalline preparations of biological macromolecules. Absent new crystallization strategies that markedly increase the order of protein crystals or modeling methods that deconvolute this disorder into multiple structural states, it appears unlikely that median resolution of MX structures in PDB will improve substantially, if at all. Fortunately for most PDB data consumers, 2 Å resolution usually suffices to reveal features of macromolecules relevant for understanding biological phenomena in 3D. In contrast, higher resolution studies may be required to understand fully biochemical functions of proteins and nucleic acids (e.g., reactions catalyzed by protein enzymes and ribozymes).

Geometric validation of atomic coordinates deposited to the PDB was introduced in the 1990s. Validation of 3D structures vs. experimental structure factors was not routinely performed until 2008, when deposition of experimental structure factor data became mandatory at the behest of the MX community. Stakeholder recommendations regarding some additional means of validating MX structures were subsequently provided in 2011 by the wwPDB X-ray Validation Task Force [53] and implemented in wwPDB legacy deposition systems in 2013 before the wwPDB global OneDep system was launched in 2014 [44]. Availability of experimental data has enabled systematic validation of atomic structures and contributed to development of better validation tools [45] and improved quality of the archived data [54].

Notwithstanding numerous aspects of 3D structure validation initially implemented within the wwPDB OneDep software system validation module, ligand validation was somewhat limited at the outset. The 2016 wwPDB/CCDC/D3R Ligand Validation Workshop recommended best practices for validation of MX co-crystal structures [55]. These recommendations were subsequently incorporated into the OneDep validation module to provide “Buster-like” 2D geometry quality and 3D electron density graphical overlays with small-molecule ligands [46]. Validation of PDB MX structures was further enhanced with introduction of uniform representation for carbohydrates [56].

Arguably, one of the most exciting new methods for measuring diffraction data at the time of writing is serial crystallography [57,58,59]. This approach is being used to probe dynamic properties of proteins and nucleic acids and visualize progress of chemical reactions in 3D (e.g., *M. tuberculosis* β-lactamase (BlaC) inactivating the β-lactam antibiotic ceftriaxone: PDB IDs 6b5x, 6b5y, 6b6a-6b6f, 6b68, and 6b69 [60]). Both X-ray free-electron lasers (XFELs) and 3rd generation synchrotron sources are being used to conduct such experiments. As of mid-2022, PDB archival holdings included 587 serial crystallography structures, with 343 (~58%) coming from XFELs and 244 (~42%) based on data collected from synchrotrons. Additionally, 217 PDB MX structures were determined using XFEL data without recourse to serial methods (e.g., PDB ID 3pcq [61]).

### 2.4. 3D Electron Microscopy (3DEM)

**Figure 5:**
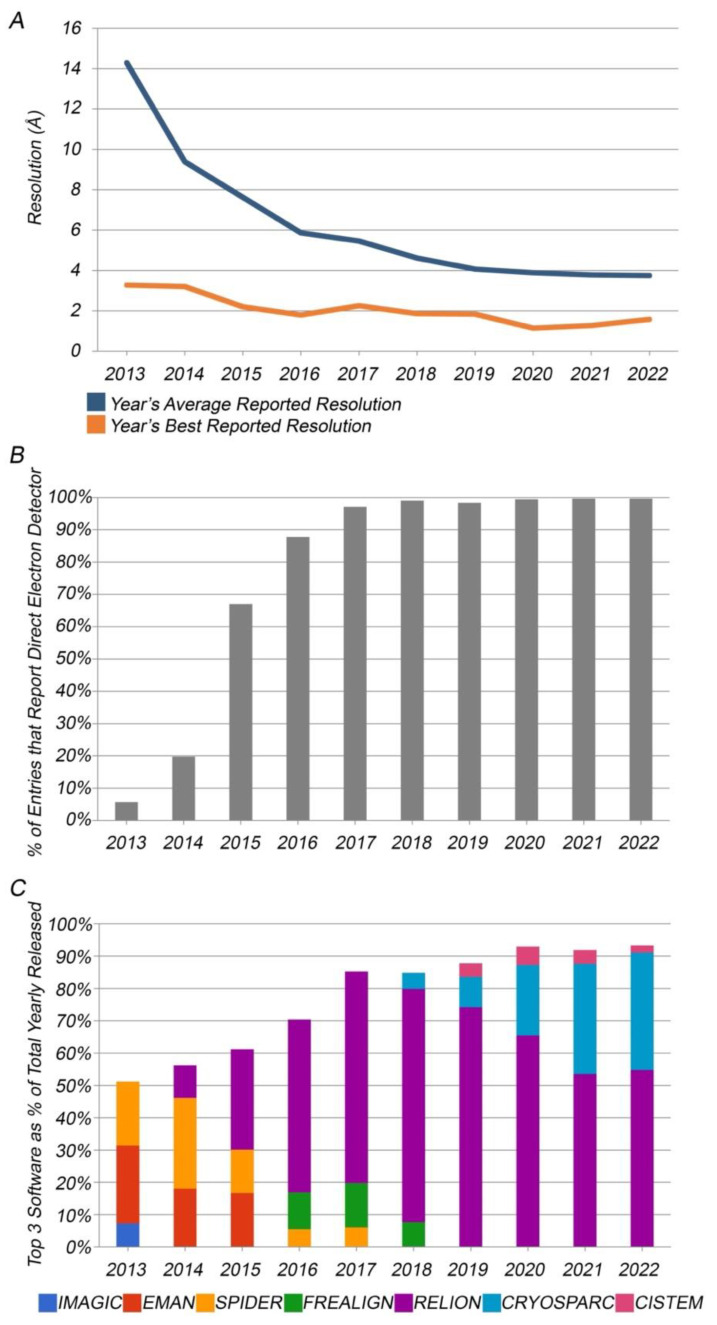
(**A**). Annual average reported resolution (blue) and annual best reported resolution (orange) for 3DEM PDB structures released 2013–2022. (**B**). Percentage of 3DEM PDB structures released per year reporting use of direct electron detectors. (**C**). Top-three reported image reconstruction software packages per year shown as a percentage of 3DEM PDB structures reporting reconstruction software.

Over the last decade, resolution of 3DEM PDB structures has improved dramatically. Since 2013, average resolution of a 3DEM PDB structure has improved from worse than 14 Å to better than ~4 Å (Figure 5A). These overall statistics, however, obscure some of the most impressive recent developments in 3DEM. Between the beginning of 2019 and mid-2022, 40 3DEM structures with resolution better than 2.0 Å were publicly released by the PDB.

Technical breakthroughs in four critical areas were responsible for this “Resolution Revolution” [62,63]. First, improvements in electron optics, driven by the needs of materials scientists and the semiconductor industry, ensure that state-of-the-art transmission electron microscopes (TEM, e.g., Thermo-Fisher Titan Krios, Waltham, MA, USA) preserve phase information at atomic resolution. Second, vitrification of biological samples and imaging under cryogenic conditions is now routine [64]. Third, direct electron detectors (DEDs) have revolutionized how we collect TEM data for single particles arrayed on EM grids. The move away from charge-coupled device (CCD) detectors to DEDs has been nothing short of a stampede. Figure 5B illustrates the trend. In 2013, only ~5% of new 3DEM PDB structures relied on DEDs. By 2017, the fraction relying on DEDs exceeded 90%, and in 2021 the fraction was ~99%. In aggregate, DEDs have been used to collect data for 10,406 3DEM PDB structures released as of mid-2022 (vs. 11,309 total 3DEM PDB structures). Finally, the other key contributor to the rapid rise of 3DEM has been advances made in data processing software. Key software engineering developments include beam-induced motion correction [65,66,67] and use of Bayesian maximum-likelihood statistics [68]. Figure 5C shows that the most popular 3DEM reconstruction software package at the time of writing is RELION [69], which has been used for determination of more than 4000 3DEM PDB structures since 2013.

Year-on-year growth of 3DEM PDB structure depositions evident in Figure 3B was driven by the single-particle method, which is revealing structures of ever more complex macromolecular assemblies and illuminating important areas of biology (e.g., ion channels, transcription–translation expressome complexes, nuclear pore complexes). Arguably even more exciting advances are yet to be made using cryo-electron tomography (cryo-ET) combined with sub-tomogram averaging [70]. One of the earliest cryo-ET structures in the archive is PDB ID 4bzj (40 Å resolution COPII Transport-Vesicle Coat Assembled on Membranes [71]). As of mid-2022, the highest resolution cryo-ET structure in the archive was PDB ID 7zbt (3.3 Å resolution RuBisCO visualized within native *Halothiobacillus neapolitanus* carboxysomes [72]). At better than 3.5 Å resolution, both α-helix and β-strand secondary structural elements and bulky amino acid sidechains are discernible in experimental 3DEM density maps (deposited to EMDB) revealing molecular details in 3D important for understanding biochemical and biological function.

The *H. neapolitanus* RuBisCO cryo-ET structure employed a relatively new sample preparation technique that relies on cryogenic dual-beam focused ion beam/scanning electron microscopes (cryo-FIB/SEM) to generate 10–20 nm thickness *lamellae* of vitrified samples using the focused ion beam to “mill” away unwanted parts of the sample. This tool allows researchers to isolate thin wafer-like volumes from inside frozen cells for subsequent cryo-ET imaging and sub-tomogram averaging. Immediate-term prospects for cryo-ET plus cryo-FIB/SEM milling with sub-tomogram averaging brightened considerable with the advent of AlphaFold2 [73,74,75] and RoseTTAFold [76]. For example, in 2021, computed structure models of human nuclear pore complex (NPC) proteins from AlphaFoldDB were combined with cellular cryo-ET and molecular dynamics simulations, to generate composite 3DEM density maps of the human NPC in both dilated and constricted conformations (PDB IDs 7r5k, 7tbl, 7tbm, 7tbj, 7tbk, and 7tbi [77]). Combining cryo-FIB/SEM with correlative light microscopy prior to cryo-ET imaging of *lamellae* holds the promise of improving the efficiency of the method by maximizing the number of molecular assemblies of interest present in a given wafer-like sample for imaging and subsequent sub-tomogram averaging [78].

At the time of writing, wwPDB validation reports for 3DEM structures included: (a) assessment of model geometry similar to that used for all MX and NMR structures (ClashScore, Ramachandran outliers, Sidechain outliers, nucleic acid polymer backbone); (b) orthogonal projections of map and map-model overlays; (c) half-map FSC plot based on mandatory half-maps collected at deposition; (d) voxel-value distribution and volume-estimation graph; (e) evaluation of map-model fit via atom-inclusion plot and residue inclusion analysis; and (f) finer evaluation of map-model fit incorporating both overall and per residue Q-scores [79]. EMDB also provides 3DEM density map and structure quality assessments on its website, including Q-scores [80]. (For more details regarding the history of 3DEM validation in the PDB, see [81]).

### 2.5. Nuclear Magnetic Resonance (NMR) Spectroscopy

Solution nuclear magnetic resonance (NMR) spectroscopy can be used to determine 3D structures of biomolecules (e.g., [82,83]). The first NMR structure of a protein was deposited to the PDB in 1988 and released publicly in 1989 (PDB ID 1bds [84]). By the end of the 1980s, solution NMR structures of 10 proteins had been determined, for which no crystallographic data were previously available [85]. At the same time, heteronuclear 3D and 4D NMR experiments were introduced to overcome limitations of spectral complexity and increased molecular weight (polypeptide chains longer than 150 amino acid residues, hereafter residues) [86]. At the beginning of the 1990s, the first NMR data file that included NMR restraints used to determine the 3D structure of Interleukin-8 (IL-8/NAP) was deposited to the archive (PDB ID 1il8 [87]). At the end of the 1990s, the first chemical shift file (containing a total of 179 chemical shifts) was deposited as part of PDB ID 1qlo [88]. Upon the recommendation of the wwPDB NMR Validation Task Force (NMR-VTF), NMR PDB structure depositions were required to include NMR restraint data and chemical shift data, in 2008 and 2010, respectively [89].

**Figure 6:**
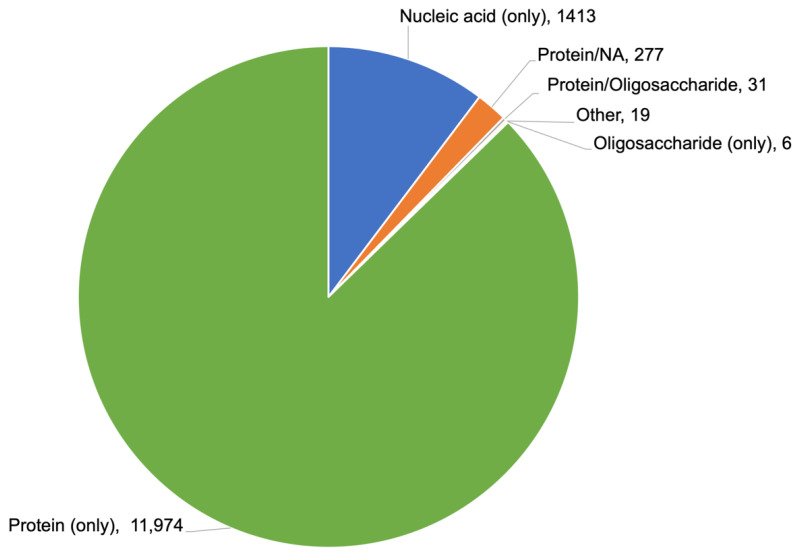
Breakdown of NMR PDB structure holdings by sample type.

The number of new NMR structures released to the public annually from the PDB peaked in 2007 at 965, when NMR structures accounted for ~17% of the entire archive. Annual depositions have been trending downward ever since (362 NMR structures released publicly in 2021), and NMR structures now account for only ~7% of PDB holdings. As of mid-2022, the archive housed 13,733 NMR structures, 13,602 solution plus 131 solid-state. Figure 6 provides a breakdown of NMR PDB structures as a function of biomolecule sample type.

Historically, NMR structural studies of biomolecules were size-limited. Most NMR PDB structures are those of smaller proteins or isolated protein domains (polymer entities < 8.5 kDa). Both solution and solid-state NMR (SSNMR) can, however, be used to study larger, more complex structures. SSNMR has been utilized to overcome some of the obstacles restricting the purview of solution NMR (e.g., relatively insoluble proteins). Both techniques can be deployed in tandem to overcome respective limitations. As of mid-2022, the PDB archive housed at least six structures determined using a combination of solution and SSNMR (e.g., *O. cuniculus* phosphorylated phospholamban homopentamer PDB ID 2m3b [90]).

Advances in technology for both solution and SSNMR have allowed for larger structures to be determined. For example, the largest solution NMR structure in the archive (as judged by total number of residues) is the Box C/D enzyme, a multimeric complex consisting of four instances of three unique proteins totaling 3044 residues (PDB ID 4by9 [91]). Additionally, use of magic angle spinning (MAS) SSNMR has enabled determination of structures with no inherent molecular size limitation, overcoming obstacles faced by solution NMR and MX. Exploiting these capabilities, SSNMR has been used to elucidate structures of complex assemblies similar in size to those studied by cryo-EM while in their native state, without the need for cryogenic preservation. As of mid-2022, the largest macromolecular structure determined by MAS SSNMR is the HIV-1 Capsid Tube, containing 378 repeats of a 231-residue subunit for a total of 87,318 residues (PDB ID 6x63 [92]). Larger structures have also been determined using integrative or hybrid methods, including that of a 484.61 kDa, 24mer αB-crystallin oligomer (4200 residues), incorporating experimental data from solution NMR, solution scattering, and 3DEM (PDB ID 3j07 [93]), and that of the 470.42 kDa tetrahedral aminopeptidase TET2 (4236 residues total), incorporating data from SSNMR and 3DEM (PDB ID 6r8n [94]).

With use of membrane-mimicking systems (e.g., micelles, bicelles, and nanodiscs), it is possible to study integral membrane proteins in their near-native environments using NMR [95]. A structure of the 7.77 kDa transmembrane domain of bacterioopsin (residues 1–71) was determined using solution NMR by solubilizing the protein in methanol/chloroform and SDS micelles, and deposited into PDB in 1993 (PDB IDs 1bha and 1bhb [96]). At the time of writing, the largest membrane protein structure determined via solution NMR deposited to the PDB is that of 149.16 kDa, 1360 residue human α7 nicotinic acetylcholine receptor, determined by a combination of solution NMR, electron spin resonance spectroscopy, and Rosetta calculations (PDB ID 7rpm [97]). As of mid-2022, the largest membrane protein structure determined by SSNMR in the PDB is that of 183.51 kDa, 1750 residue M13 bacteriophage capsid (PDB ID 2mjz [98]).

In addition to the study of 3D structures of biological macromolecules, examination of dynamics is often important for understanding function. Insights into a biomolecule’s local dynamic behavior can be used to identify parts of structures important for ligand binding, protein–protein or protein–nucleic acid interactions, allostery, or conformational changes (e.g., integral membrane proteins). NMR spectroscopy is uniquely capable of studying macromolecular movement because of its ability to study samples spanning a wide range of solvent/solute conditions at atomic resolution over relevant timescales (i.e., picoseconds to seconds). Such studies are also possible using MAS SSNMR, which can be used to interrogate dynamics of the protein backbone atoms and sidechains (both globally and locally). As of mid-2022, the PDB archive housed results of dynamics studies of both small proteins (e.g., 8.58 kDa ubiquitin, PDB ID 2k39 [99]) and large biological nanomachines (e.g., 181.87 kDa proteasome subunit alpha heptamer, PDB ID 2ku1 [100]).

As is the case for MX and 3DEM, validation standards for NMR structures archived in the PDB are being developed collaboratively by the wwPDB and independent experts. Following implementation of chemical shift validation in 2015 at the behest of community stakeholders, the NMR Data Exchange Format (NEF) Working Group, which includes developers of NMR structure determination and refinement software packages, recommended use of a common exchange format to represent NMR chemical shifts, restraints, and related metadata [101]). NMR structure validation utilizing this unified exchange format was incorporated within the wwPDB OneDep software system and wwPDB validation reports in 2020. At the time of writing, archive-wide regeneration of extant NMR structure validation reports to enable restraint validation was underway. Completion of this remediation project and public release of regenerated wwPDB validation reports for all NMR structures archived in the PDB is anticipated in 2023. Additional improvements in wwPDB validation of NMR structures is expected to encompass data representation and validation of multiple conformers (e.g., pro-islet amyloid polypeptide open conformer (PDB ID 6ucj) and pro-islet amyloid polypeptide bent conformer (PDB ID 6uck [102]) and validation of structures determined using NMR combined with other experimental methods (e.g., PDB ID 3j07 [93]).

### 2.6. Electron Crystallography (EC) and Micro-Electron Diffraction (microED)

Electron diffraction or electron crystallography (EC) has also been used to determine 3D structures of biological macromolecules. The method employs 2D crystals, beginning with those of bacteriorhodopsin, the first integral membrane protein structure to be deposited into the archive (PDB ID 1brd [103], resolution 3.5 Å). Prior to 2013, a total of 37 biostructures determined using EC were deposited to PDB. With the advent of modern electron microscopes, a new electron diffraction method using miniscule 3D crystals (micro-electron diffraction or microED) has been developed [104]. The first microED structure of a globular protein (hen egg white lysozyme, PDB ID 3j4g [105], resolution 2.9 Å) was deposited to the PDB in late 2013. As of mid-2022, the PDB housed 137 microED structures of biomolecules, the largest two of which are human adenosine receptor A2a/cytochrome b562 chimeric protein (PDB ID 7rm5, 50 kDa, resolution 2.8Å [106]) and bovine catalase (PDB ID 3j7b, 60 kDa, resolution 3.2Å [107]). Unlike most EC structures archived in PDB, microED structures are typically determined at very high resolution. As of mid-2022, the highest resolution microED structure in PDB was that of hen egg white lysozyme (PDB ID 7skw [108], resolution 0.87 Å).

### 2.7. PDB Archive Management and Weekly Update/Release

The PDB data standard is defined by the PDBx/mmCIF dictionary [109,110,111]. It is the macromolecular extension of an earlier community data standard, the Crystallization Information Framework (cif.iucr.org, accessed on 28 August 2022), developed for small molecules by the International Union of Crystallography [112]. The macromolecular data standard is maintained by the wwPDB partnership together with the wwPDB PDBx/mmCIF Working Group (wwpdb.org/task/mmcif, accessed on 28 August 2022) [111]. wwPDB partners and the Working Group collaborate on developing terminologies for new and rapidly evolving methodologies and remediating (or enhancing) representations for existing data content.

In its role as wwPDB-designated PDB Archive Keeper, RCSB PDB is responsible for safeguarding >100 TB of digital information and a physical archive that includes correspondence and other archive-related artifacts dating back to the early 1970s. Snapshots of the digital information are preserved annually and following large-scale archive-wide data remediation campaigns, the most recent of which involved standardizing atom naming, etc. for >14,000 carbohydrate-containing structures in the PDB [56]. The size of the 2021 digital snapshot was ~1 TB, which does not include ~4.5 TB of 3DEM density map information archived in EMDB (also jointly managed by the wwPDB partnership).

In its role as wwPDB-designated Archive Keeper, RCSB PDB is responsible for weekly updates of the PDB archive using the following two-stage process:

**Stage One** releases sequence(s) for each distinct polymer (amino acid or nucleotide) in the structure; InChI string(s) for each distinct ligand; and crystallization *p*H value(s), where appropriate, on the wwPDB web portal (see www.wwpdb.org/ftp/pdb-ftp-sites, accessed on 28 August 2022) every Saturday by 03:00 Universal Time Coordinated (UTC). This first stage in the process supports weekly blind challenges for in silico prediction of protein structure (CAMEO, cameo3d.org, accessed on 28 August 2022 [113]) and small-molecule docking (CELPP, drugdesigndata.org/about/celpp, accessed on 28 August 2022 [114]).

**Stage Two** completes the weekly process every Wednesday at 00:00 UTC by releasing the updated PDB archive in full (currently adding ~300 new structures/week, updating previously released structures with literature citation information, etc., and on occasion removing obsolete structures).

PDB data are freely distributed online, providing universal open access to the archival information in two forms (latest archive, files.wwpdb.org/pub/pdb/data, accessed on 28 August 2022; and latest and prior versions of archive, files-versioned.wwpdb.org, accessed on 28 August 2022). Hypertext Transfer Protocol (HTTP) and remote sync (rsync) are recommended for access; File Transfer Protocol (FTP) access will be retired in late 2024. PDB data are also made available without storage fees or egress charges by Amazon Web Services (AWS) through its Open Data Sponsorship Program (registry.opendata.aws/pdb-3d-structural-biology-data/, accessed on 28 August 2022).

Global PDB archive data downloads in 2021 reached a record high of 2,364,150,827 structure data files, which represents an ~80% increase vs. the previous record of 1,323,213,832 set in 2020. Approximately 70% of global structure data file downloads in 2021 originated from the FTP archive. The remainder were accessed by users of wwPDB member web portals.

### 2.8. All Three Kingdoms of Life Are Represented in the PDB Archive

**Figure 7:**
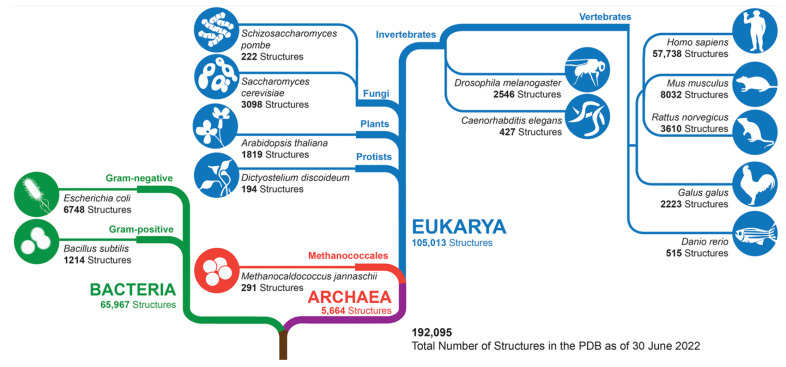
Phylogenetic Tree showing PDB holdings (as of mid-2022). Within each of the three branches, PDB structure totals are provided for selected organisms. N.B.: The PDB also houses 3D structures that solely contain nucleic acids (DNA, RNA, DNA-RNA hybrids, etc.) and/or viral proteins or human-designed proteins, which collectively accounted for ~8% of archival holdings as of mid-2022.

As of mid-2022, MX, 3DEM, NMR, EC, and microED had been used collectively to determine >190,000 3D biostructures housed in the PDB archive, which encompasses proteins from organisms representing all living kingdoms (Figure 7). Archaebacterial proteins were the least numerous (totaling 5664 structures), followed by bacteria (65,967 structures). PDB holdings of eukaryotic protein structures exceeded 105,000, with more than half being human in origin. There is limited PDB coverage across the so-called model organisms, with mouse proteins being most numerous at >8000 structures.

### 2.9. PDB Data Delivery/Usage Metrics

**Figure 8:**
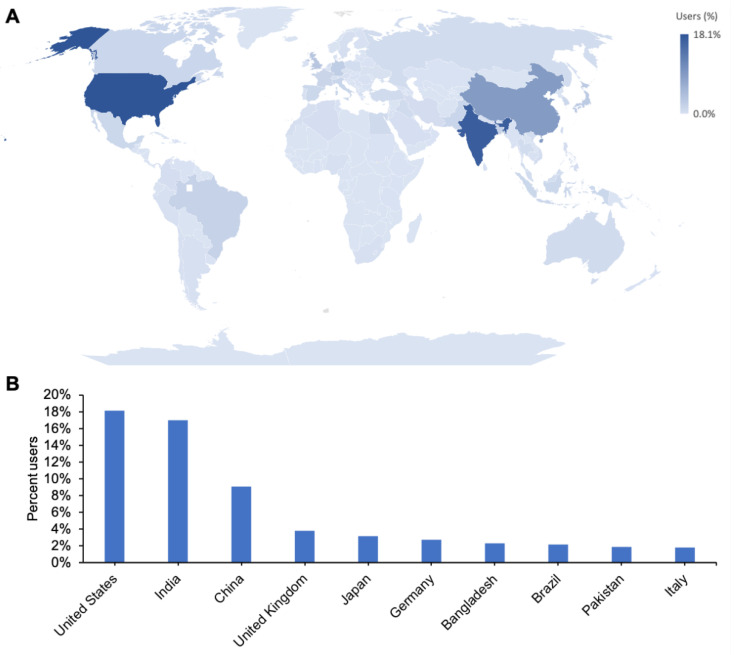
(**A**). Geographic distribution of RCSB.org users by country. (**B**). Top 10 countries with the highest percentage of users from 2019–2021. Data from Google Analytics.

Most RCSB PDB users access the archive through our RCSB.org research-focused web portal, which makes PDB data available at no cost with no limitations on usage via the Creative Commons CC0 1.0 Universal license (creativecommons.org/publicdomain/zero/1.0/, accessed on 28 August 2022). In 2021, 6,845,233 unique internet protocol (IP) addresses from more than 240 countries and territories recognized by the United Nations (Figure 8A) were used to access RCSB.org (exceeding the 2020 pandemic lock-down record of 6,677,853). Figure 8B ranks RCSB.org utilization for the top ten user countries for 2019–2021. Not surprisingly, the US–RCSB PDB’s host country–has the largest percentage of users, followed by the world’s two most populous nations, India and the People’s Republic of China.

We estimate that ~99% of PDB data consumers are not experts in structural biology. Their research interests are extremely broad, encompassing fundamental biology, biomedicine, energy sciences, bioengineering, and biotechnology [115,116]. Beyond the natural, physical, mathematical, and engineering sciences, there is also use of PDB data by social scientists (e.g., economists, [117,118]).

The RCSB.org web portal provides added value to PDB users that goes well beyond the content of the archive itself. On a weekly basis, RCSB PDB integrates PDB data with information from ~50 trusted external resources (Table 1). Integrating individual PDB structures with information from trusted external resources ensures that the RCSB.org web portal operates as a “living data resource.” Scholarly journal articles describing PDB structures are static documents, reflecting what was known about the biomolecule(s) at the time of publication. Thereafter, it is not uncommon for new biological or biochemical functions of a macromolecule to come to light, or new disease-causing mutations to be identified. Such new findings are integrated with PDB data every week, thereby ensuring that RCSB.org users have access to the most current information pertaining to every 3D biostructure in the public domain.

PDB data utilization worldwide is also mediated by third parties that repackage and reuse the archival information. While the RCSB PDB is unable to assess utilization of the archive via third parties, review of the Nucleic Acids Research Online Molecular Biology Database Collection [156], which comprises databases from *Nucleic Acids Research* annual Database Issues, identified 460 external data resources that distribute repackaged PDB data (Supplementary Materials Table S1). Additional utilization of PDB data occurs within all major biopharmaceutical companies and many smaller biotechnology companies that maintain copies of the archive inside company firewalls. They frequently use PDB data alongside proprietary MX structures determined by company structural biologists or their contractors. Most, if not all, global biopharmaceutical companies (e.g., Pfizer, Novartis, Eli Lilly and Company) rely on structure-guided drug discovery of small-molecule, orally bioavailable therapeutic agents, which typically begins with scanning of PDB archival holdings for a public domain structure of the target protein to begin the discovery process [25,157,158]. They also make use of PDB structures when engineering new biologic agents (monoclonal antibodies, cytokines, etc.) for use as injectables [159].

Literature searching provides another means of assessing utilization and impact of PDB data. As of mid-2022, 162,262 (~84%) of PDB structures are described in 75,497 unique primary publications, the vast majority of which appeared in peer-reviewed journals. Citation analyses carried out using EuropePMC revealed that in 2021, the PDB was mentioned by name in 23,030 publications. It further documented that PDB IDs were mentioned in 585,903 publications in 2021. An RCSB PDB study published in 2018 [160] documented that citations of PDB data spanned the sciences, literally from Agriculture to Zoology. Not surprisingly, nearly 90% of published PDB structures analyzed in 2018 were cited by journals in the area of Biochemistry and Molecular Biology. High impact within other areas of biomedicine (Cell Biology, Pharmacology and Pharmacy, Microbiology, Genetics and Heredity) was, as expected, also documented. Further RCSB PDB analyses on this topic highlighted PDB structure publications that were frequently cited in scientific journals focused on Materials Science, Physics, Computer Science, Chemistry, Engineering, and Mathematics [116].

Searching of the patent literature in August 2022 also documented substantial impact of PDB data. Directed searches for PDB mentions using the US Patent and Trademark Office website (uspto.gov, accessed on 28 August 2022) identified nearly 19,000 in-process patent applications and ~10,000 issued US patents (vs. ~20,000 in process applications and ~6500 issued patents in June 2017 [160]). Analogous searches of global patent literature using PatSeer (patseer.com) documented ~90,000 issued patents and patent applications in process worldwide that include PDB mentions (vs. ~50,000 in June 2017 [160]).

**Figure 9:**
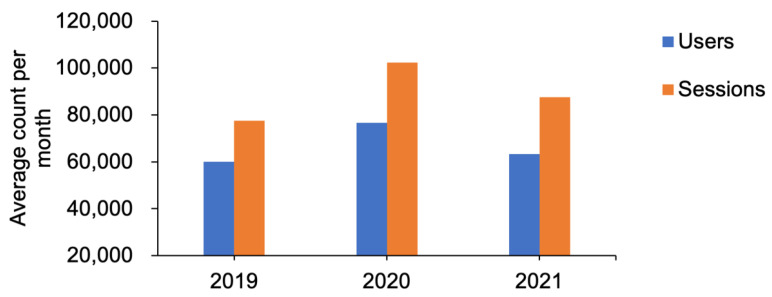
Average monthly usage of PDB-101 (PDB101.RCSB.org, accessed on 28 August 2022) from 2019–2021. Data from Google Analytics.

Finally, RCSB PDB also operates a second web portal focused on outreach and education (PDB101.RCSB.org, with PDB-101 denoting an introductory course) [161]. PDB-101 was launched in 2011 to support PDB archive exploration and training by university faculty, postdoctoral researchers, undergraduate and graduate students, school teachers and their pupils, and the general public. It was established to help train the next generation of PDB users and promote structural biology and protein science to non-experts. Regularly published features include the highly popular *Molecule of the Month* series [162], 3D biostructure-related activities, molecular animations and videos, and educational curricula, many of which are organized around a public health topic [163]. The *Guide to Understanding PDB Data* covers key topics, including file format information and explanations of the types of data included with a PDB entry. Materials are organized into various categories (Health and Disease, Molecules of Life, Biotech and Nanotech, and Structures and Structure Determination) and searchable by keyword (e.g., cancer, checkpoint therapy, antibody). Although it is not as intensively accessed as our RCSB.org research-focused web portal, there is substantial utilization of PDB101.RCSB.org by users from around the world (Figure 9).

### 2.10. Impact of PDB Data on Computational Structure Modeling

Use of PDB data to compute 3D structure information for other proteins is well-established. For many years, publicly available computational services (e.g., Modeller/ModBase [164,165,166] and ProMod3/SWISS-MODEL, [167,168] and Rosetta [169]) used comparative or homology modeling to predict protein structures. This approach depends on finding an experimentally-determined protein structure in the PDB with an amino acid sequence similar to that of the target protein to use as a modeling template or scaffold. Homology modeling typically succeeds when a structural template with >40% sequence identity is available. Like MR, homology modeling is often useful because of the parsimony of macromolecular evolution.

As the PDB archive grew, template-free computational structure modeling became possible for very small globular proteins. Continuous advances in both homology modeling and template-free protein structure prediction were fostered by two community-led blind challenges (i.e., CASP [170], and the weekly Continuous Automated Model EvaluatiOn (or CAMEO) online challenge [113]). Both CASP and CAMEO rely on coordination with structural biologists and the wwPDB to ensure relevant structure data are not publicly released before each challenge concludes.

**Figure 10:**
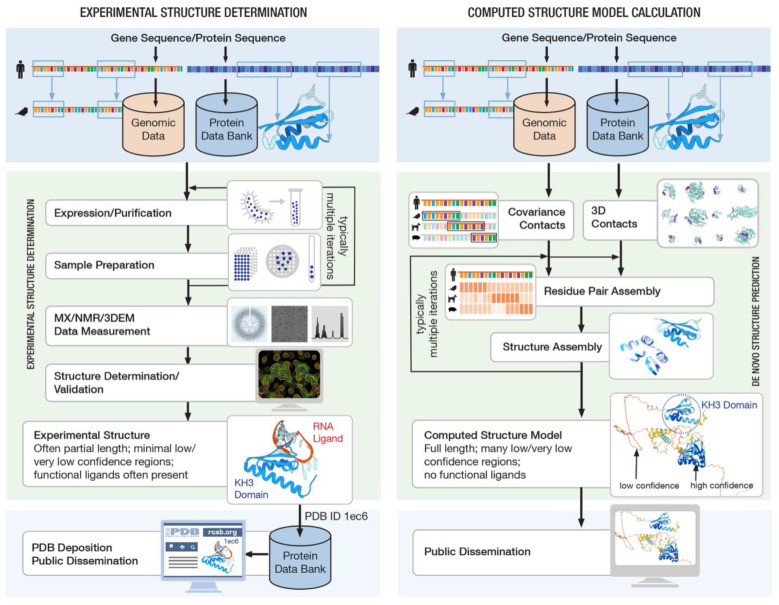
Experimental approaches for determination of protein structures and computational methods for predicting structures both rely on open access to genomic and 3D structure data. Here, methods for determining the structure of the RNA-binding protein Nova-2 are shown. The MX structure (**left**) was determined for an isolated domain of the protein bound to its RNA target. The computed structure (**right**) includes the entire polypeptide chain, which is predicted to include three well-folded domains (blue/cyan) connected by apparently unstructured linkers (yellow/orange). Image adapted from *New England Journal of Medicine*, Stephen K. Burley, Wadih Arap, Renata Pasqualini, Predicting Proteome-Scale Protein Structure with Artificial Intelligence, 385, 2191–2194 [173].Copyright © 2022 Massachusetts Medical Society. Reprinted with permission.

Google DeepMind emerged as the top performer in the 2020 CASP challenge [170]. Its AlphaFold2 software uses artificial intelligence/machine learning (AI/ML) to predict 3D structures of smaller globular proteins with accuracies comparable to that of low-resolution experimental methods [74]. It was rightly heralded as a major breakthrough in de novo protein structure prediction. Subsequently, the Rosetta team led by David A. Baker (University of Washington/Howard Hughes Medical Institute) released RoseTTAFold [76] and then RoseTTAFold2, which also use AI/ML methods to generate computed structure models (CSMs) of proteins with reported accuracies comparable to that of AlphaFold2. Figure 10 contrasts experimental structure determination with computed structure model calculation. At the time of writing, CSMs for nearly every protein sequence represented in UniProt [155] generated by DeepMind using AlphaFold2 were publicly available from AlphaFold DB [73,74,75]. Some of the CSMs generated by computational biologists operating independently of DeepMind (using RoseTTAFold, AlphaFold2, etc.) are available from the open access ModelArchive (modelarchive.org, accessed on 28 August 2022).

Of particular importance when evaluating CSMs for use in research are pLDDT (predicted local distance difference test) scores or confidence estimates generated by AlphaFold2 [74,171]. pLDDT scores (scaled between 0 and 100) denote polypeptide chain segments as very high confidence (pLDDT ≥ 90), confident (90 > pLDDT ≥ 70), low confidence (70 > pLDDT ≥ 50), and very low confidence (pLDDT < 50). We do not yet know how much enhanced AI/ML methods will improve prediction accuracy and expand the scope thereof to larger, multidomain proteins, but history shows us that continued growth of the PDB should only help in this regard.

It is no exaggeration to say that neither AlphaFold2 nor RoseTTAFold2 would exist today without open access to complete, rigorously validated, expertly biocurated 3D biostructure data from the PDB [172]. Looking ahead, use of AI/ML methods for accurate prediction of structures of macromolecular assemblies and, perhaps even more challenging, transient intermolecular interactions that underpin complex regulatory processes in biology will depend critically on continued growth in the number of 3DEM structures of large molecular machines deposited to the PDB. Successful application of AI/ML methods for predicting small-molecule ligand binding to protein targets may not be possible in the near term given current PDB data deposition trends. The number of co-crystal structures of small molecules binding to proteins in the PDB is dwarfed by 3D structure data collectively held as trade secrets across the biopharmaceutical industry. Contributions of significantly more co-crystal structure data from industry would almost certainly fuel advances in prediction of small-molecule binding to proteins. With sufficient data placed in the public domain, we can reasonably expect that AI/ML methods would accelerate drug discovery and development efforts in both academe and industry for the greater good [172].

### 2.11. Future Directions

The futures of structural biologists and the PDB appear even brighter, contrary to post-AlphaFold2 rumors to the effect that experimental structural biology is on the verge of precipitous decline. Depositions of structures to the PDB in 2022 are on track to exceed those in all previous years. Experimentally determined 3D biostructures are highly prized accomplishments. Medium-to-high resolution experimental structures (e.g., MX structures better than 3.5 Å resolution) are more accurate than CSMs [174]. Moreover, they frequently contain bound small-molecule ligands of biological or biomedical importance. They may also include more than one macromolecule, providing information regarding homo- and hetero-meric assemblies that underpin the workings of complex molecular machines.

CSMs generated with AI/ML methods are of considerable interest to experimental structural biologists. Many are taking a “glass half full” approach to this information. They often rely on CSMs of large multi-domain eukaryotic proteins for designing protein expression constructs by excluding low confidence and very low confidence regions when generating truncations suitable for MX, NMR, or 3DEM studies. (N.B.: CSMs are not eligible for archiving in the PDB, because they do not involve measurements from a sample of the biological macromolecule for which the structure is determined.)

The future of experimental structural biology is also looking bright. Researchers are tackling ever larger and more complex macromolecular machines using so-called integrative or hybrid methods that combine experimental measurements from more than one biophysical technique. Anticipating this trend, a wwPDB Integrative/Hybrid Methods (IHM) Task Force was assembled to make recommendations regarding data archiving and structure validation [175,176]. As an interim measure, the wwPDB established PDB-Dev as a standalone prototype system [177,178,179] for archiving and publicly disseminating integrative structures and associated data. Integrative structure determination entails making measurements using complementary experimental methods (e.g., 3DEM and chemical cross-linking) and converting the results into spatial restraints that are applied to with known starting structures of molecular components to determine the structures of complex macromolecular assemblies.

The PDB-Dev software system supports data collection, processing, curation, validation, archiving, and distribution of integrative biostructures. It is underpinned by ModelCIF (github.com/ihmwg/ModelCIF, accessed on September 29 2022), an expanded set of data standards based on the PDBx/mmCIF data standard (above) for representing integrative structures and associated experimental restraints; a software library that supports the new data standards; a data harvesting system for collecting heterogeneous data from diverse experimental techniques, methods for curating, validating and visualizing integrative structures; and web services for distributing archived data. The PDB-Dev prototype system has allowed structural biologists to make their integrative structures publicly available, including but by no means limited to those involved in transport of proteins and nucleic acids across the nuclear envelope (nuclear pore complex [180]), regulation of gene expression (expressome complex [181]), cellular vesicle trafficking (exocyst complex [182]), and regulation of genomic architecture (BAF complex [183]). Importantly, the PDB-Dev data standard was designed to interoperate with PDBx/mmCIF and the PDB, so that integrative structures and related metadata can eventually be archived in the PDB.

In parallel with building PDB-Dev, wwPDB partners are working to establish a federated network of interoperating structural biology data resources, as recommended by the IHM Task Force [176]. This effort involves collaboration with other experimental data repositories (e.g., SASBDB [184] and PRIDE [185]). Tools are being created to support automated data exchange between PDB-Dev and these and other biodata repositories (e.g., BioImage Archive, www.ebi.ac.uk/bioimage-archive, accessed on 28 August 2022 [186]). The overarching goal of the wwPDB partnership is to foster federation of structural biology data resources across length scales ranging from atoms to individual proteins to macromolecular machines to organelles to cells and eventually tissues to maximize the impact that atomic level 3D biostructures will have on research and education across basic and applied biological, biomedical and energy sciences.

## Figures and Tables

**Table 1:** Trusted external resources/data content integrated weekly with PDB archival data by RCSB PDB from rcsb.org/docs/general-help/data-from-external-resources-integrated-into-rcsb-pdb (accessed on 28 August 2022). (N.B.: In response to community input, RCSB PDB continues to integrate new external data resources.).

Resource	Description
AlphaFold DB [73,74]	Computed Structure Models by AlphaFold2
ATC	Anatomical Therapeutic Chemical (ATC) Classification System from World Health Organization
Binding MOAD [119]	Binding affinities
BindingDB [120]	Binding affinities
BMRB [13]	BMRB-to-PDB mappings
CATH [121]	Protein structure classification
CCDC [122]	Cambridge Structural Database (CSD)
ChEBI [123]	Chemical entities of biological interest
ChEMBL [124]	Manually curated database of bioactive molecules with drug-like properties
DrugBank [125]	Drug and drug target data
ECOD [126]	Evolutionary Classification of Protein Domains
EMDB [11]	3DEM density maps and associated metadata
ExplorEnz [127]	IUBMB Enzyme nomenclature and classification
Gencode [128]	Gene structure data
Gene Ontology [129]	Gene structure data
Genotype-Tissue Expression (GTEx) [130]	Tissue-specific gene expression data
GlyCosmos [131]	Web portal integrating the glycosciences with the life sciences
GlyGen [132]	Data integration and dissemination resource for carbohydrates and glycoconjugates
GlyTouCan [133]	Glycan structure repository
Human Gene Nomenclature Committee (genenames.org, accessed on 28 August 2022)	Human gene name nomenclature and genomic information
IMGT [134]	International ImMunoGeneTics information system
Immune Epitope Database [135]	Antibody and T cell epitopes
International Mouse Phenotyping Consortium (mousephenotype.org, accessed on 28 August 2022)	Mouse gene phenotype data
InterPro [136]	Classification of Protein Families
MemProtMD [137]	Database of Membrane Proteins Embedded in Lipid Bilayers
ModelArchive (modelarchive.org accessed on 28 August 2022)	Computed Structure Models (e.g., by RoseTTAFold)
Mpstruc [138]	Classification of transmembrane protein structures
NCBI Gene [139]	Gene info, reference sequences, etc.
NCBI Taxonomy [139]	Organism classification
NDB [140]	Experimentally determined nucleic acids and complex assemblies
OPM [141]	Orientations of Proteins in Membranes database; Classification of transmembrane protein structures and membrane segments
PDBbind-CN [142]	Binding affinities
PDBflex [143]	Protein structure flexibility
PDBTM [144]	Protein Data Bank of Transmembrane Proteins
Pharos [145]	Drug targets and diseases
ProteinDiffraction.org (proteindiffraction.org, accessed on 28 August 2022)	Diffraction images
PubChem [146]	Chemical information
PubMed [139]	Citation information
PubMedCentral [139]	Open access literature
RECOORD [147]	NMR structure ensembles
RESID [148]	Protein modifications
SAbDab [149]	The Structural Antibody Database
Thera-SAbDab [150]	Therapeutic Structural Antibody Database
SBGrid [151]	Structural Biology Data Grid/diffraction images
SCOP [152]	Structural Classification of Proteins
SCOPe [153]	Structural Classification of Proteins—extended
SIFTS [154]	Structure, function, taxonomy, sequence
UniProt [155]	Protein sequences and annotations

## Data Availability

PDB data are made freely available by the wwPDB (wwPDB.org, accessed on 28 August 2022).

## Supplementary Materials

The following supporting information can be downloaded at: https://www.mdpi.com/article/10.3390/biom12101425/s1, Table S1 Cumulative list of external data resources identified as repackaging and redistributing PDB data.
