# Supplementary material for: Protein Data Bank: A Comprehensive Review of 3D Structure Holdings and Worldwide Utilization by Researchers, Educators, and Students
Source: Biomolecules. 2022 Oct 4;12(10):1425. doi: 10.3390/biom12101425 (PMC9599165; doi:10.3390/biom12101425)
Supplement: Supplementary file 1 [file biomolecules-12-01425-s001.zip › biomolecules-1917581-supplementary.pdf]

Supplementary Table S1. Cumulative list of external data resources identified as repackaging and redistributing PDB data (Source: *Nucleic Acids Research Molecular Biology Online Database Collection*).

1-9

16S and 23S Ribosomal RNA Mutation Database

2P2Idb*

3D rRNA modification maps

3D-Footprint*

3D-Interologs

3DID - 3D interacting domains

3DSwap: Database of Proteins involved in 3D domain Swapping

A

ABCD

AcrHub

ActiveDriverDB

ADPriboDB

AffinDB*

Aging Atlas

AHTPdb

Allosteric Database

AlphaFold Protein Structure Database

AmoebaDB

AmyPro

anti-CRISPRdb

AntigenDB

APD - Antimicrobial Peptide Database

ApiDB - Apicomplexan DataBase

ApoHoloDB

APPRIS

ArachnoServer*

ArchDB

AS-ALPS

ASPD

AspGD*

ASPicDB

ATGC*

Autophagy Database

AutoPSI*

B

BACTIBASE

BAliBASE

BastionHub

BCIpep

Benchmark*

BiGG Models

BIGNASim

Binding MOAD

BindingDB

BioGPS

BioLiP

BioMagResBank

BIOZON*

BRENDA

BSDB

BSORF - Bacillus subtilis Open Reading Frames

C

CADB - Conformational Angles DataBase of Proteins*

CAMP

Cancer3D

CancerPPD

CancerResource*

CanSAR

CAPS-DB

Carbohydrate Structure Database (CSDB)*

CARD

CarpeDB

CATH

CAZy

CC+

ccPDB

CDD

CFGP

CGD - Candida Genome Database

ChannelsDB

ChEBI - Chemical Entities of Biological Interest

ChemBank

ChEMBL

ChlamDB

CIPRO

CMGSDB

CMNPD

COMBREX*

COMe - Co-Ordination of Metals etc.*

ComSin*

ConoServer

ConsensusPathDB

ConSurf-DB

COSMIC

CoV3D

CovInDB

CovPDB

CPDB

CPPsite

CryptoDB - Cryptosporidium database

CSA - Catalytic Site Atlas

CSDBase - Cold Shock Domain database

CSS - Carbohydrate Structure Suite*

Cube-DB*

Cybase

D

Dali database*

DAnCER*

DARC*

DATF

DBAASP

DBAli*

dbCAN-seq*

DBD

dbPTM

dbRIP

dbSNO*

DDBJ

Death Domain database

Decoys-R-Us*

Defensins Knowledgebase

Degradome Database

DEPOD

DIGGER

DIMA

DisProt - Database of Protein Disorder

DKK

DNAmoreDB

DNASU Plasmid Repository

DockCoV2

Dockground

DomIns - Database of Domain Insertions*

DOMMINO*

DrLLPS

Drosophila polymorphism database

DrugCentral

DrugSpaceX

DSD*

DSDBASE

DualSeqDB

E

EBI Enzyme Portal

ECMDB

ECOD

eF-site - Electrostatic surface of Functional site*

EK3D

ELM - Eukaryotic Linear Motif: functional sites in eukaryotic proteins

EMDataBank

Ensembl

Enzyme Nomenclature

Epitome

eProS*

euHCVdb

Europe PubMed Central

European Nucleotide Archive

EVEREST - EVolutionary Ensembles of REcurrent SegmenTs*

ExplorEnz

Exposome-Explorer

EyeSite*

EzCatDB

F

Factorbook

FAIRDOMHub

FANTOM

FireDB

FireProtDB

FlyMine

fPOP

FragmentStore*

FungiDB

FuzDB

G

G4LDB

Gene Wiki

Gene4Denovo

GeneCards

Genome3D*

Genomic Threading Database*

GermOnline

GiardiaDB.org

Glycan Fragment DB

GlycomeDB

GlyTouCan

GPCRDB

gpDB - G-protein database

GproteinDb

GTOP - Genomes To Protein structures

GWIDD

GyDB

H

H-Invitational Database, an integrated database of human genes and transcripts

HCAD - Human Chromosome Aberration Database*

HCV Database

Heme Protein Database

Hemolytik

Het-PDB Navi

HIC-Up*

HIV RT and Protease Sequence Database

HMDB - The Human Metabolome Database

HMPD*

HOMSTRAD - Homologous Structure Alignment Database

Hoppsigen

HotRegion*

HotSprint*

HPID - Human Protein Interaction database

HPRD - Human Protein Reference Database

HRaP - Database of occurrence of HomoRepeats and Patterns in proteomes

I

IBIS

IDDB

IDEAL

IEDB

IEDB-3D*

IGDD*

iGNM

IMGT/3Dstructure-DB

IMGT/mAb-DB

IMOTdb

INDI

Influenza Research Database

InnateDB

IntEnz

Inter-Chain Beta-Sheets*

InterEvol

InterRNA

iProClass

IUBMB Nomenclature database

IVDB - Influenza Virus Database*

J

JAIL*

JASPAR

Jenalib: Jena Library of Biological Macromolecules

JET2 Viewer

K

KBDOCK*

KIDFamMap

KinaseMD

Kincore

KLIFs

KnotProt

Knottin database

L

Laminin Database

LenVarDB

LigAsite*

LinkProt

Lipase Engineering Database

Supplementary Table S1, cont.

LitCovid
LOCATE*
LOX-DB

M

MACiE
MALISAM
markerDB
MatrixDB
MegaMotifbase
Membranome
MemMoRF
MeMotif*
MEROPS
MetaBioME*
MetaCyc
MetalPDB
metaTIGER
MHCBN
MicrobesOnline
MicrosporidiaDB
MimoDB
MINAS
MIPModDB
MitoProteome
MMCD
MMDB*
MMsINC
MobiDB
ModBase
MODOMICS
MoKCa
MolMovDB - Database of Macromolecular Movements
MoonProt
Mouse Genome Database (MGD)
MP:PD*
MPDB - Molecular Probe Database
MulPSSM
MultiTaskDB
MutDB*
mutLBSgeneDB

N

Nanobase.org
NBDB
NCBI BioSample/ BioProject
NCBI BioSystems*
NCBI Protein database
NDB
Negatome
NetworKIN
neXtProt
NLSdb
NMPdb - Nuclear matrix associated proteins database
NONCODE
NORINE
NP-MRD
NPIDB (Nucleic acids & Protein Interaction DataBase)
NURSA

O

O-GLYCBASE
OMPdb*
ONQUADRO
OnTheFly
Ontobee
Open Targets
OPM
ORENZA*

P

P3DB
PAMDB
Papillomavirus Episteme
PASS2
Pathguide
PATRIC, the bacterial bioinformatics database and analysis resource*
PCDDB
PCOSKB
PCRPi-DB
PDB facilities*
PDB_TM
PDBe
PDBflex
PDBj
PDBSite
PDBsum
pE-DB*
PEDANT*
Peptaibol
Pfam
PharmGKB
Pharos
PhaSepPro *
PhosphoSItePlus
PhytAMP
PIR - Protein Information Resource
piSite
Plant Reactome
PlantTFDB
Platinum*
PLPMDB*
PMAP*
PMDB - Protein Model Database*
PncsHub
Pocketome*
Polbase*
PortEco
PoSSuM
PPD
PPT-DB
prePPI
PREX
PRIDE
proCHiPdb
ProDom*
ProGlycProt
PROMISCUOUS*
ProNAB
ProSAS*
PROSITE
PROTAC-DB
ProtChemSI
ProtCID
Protegen*
Protein kinase resource*
Protein-protein interfaces
Proteome-pI
ProteomeScout
ProTeus*
ProTherm
ProtoNet*
ProtozoaDB*
PSCDB
Pseudomonas Genome Database
PTGL
PubChem
PyIgClassify

R

R.E.DD.B.
RAID*
RAPID
RCSB Protein Data Bank
Reactome
RegulonDB now RegulonDB v 10.5
REPAIRtoire
RepeatsDB
Rfam
Ribonuclease P Database*
RNA Bricks
RNA CoSSMos*
RNA FRABASE
RNA SSTRAND
RNAcentral
RNAJunction
RNApathwaysDB*
RNArchitecture
RsiteDB

S

SAbDab
SASBDB
SATPdb
sc-PDB
SchistoDB*
SCOP2 - Structural Classification Of Proteins
SCOPe
SCOPPI
SCoV2-MD
SDAP
SecReT4
SFLD
SGD - Saccharomyces Genome Database
SIFTS
SISYPHUS*
SitEx*
SMART
SMoS.2: Update of database of Structural Motifs in Superfamilies
SNAPPI
SNPeffect
SoyKB - Soybean Knowledge Base
Spliceosome Database
SPROUTS*
SRPDB*
SSToSS - Sequence-Structural Templates of Single-member Superfamilies
STAP refinement of NMRdb*
Start2Fold
STCRDab
STINGreport
STITCH
STRING
Structure Superposition Database
SubtiWiki
SugarBindDB
SuperCYP*
SuperDrug*
SUPERFAMILY
SuperPain*
SuperSweet*
SuperTarget*
SuperToxic*
SWEET-DB
SWISS-2DPAGE
SWISS-MODEL Repository
SYNBIP
SynSysNet*
SZGR

T

T-psi-C
T3DB
TAED - The Adaptive Evolution Database
Target-Pathogen
TCDB
Telomerase database
TFClass
The Complex Portal
ThermoMutDB
ThYme
tmRDB
TopDB
TopoSNP
ToxoDB - The Toxoplasma gondii Genome Database
Transformer*
TriTrypDB
TSTMP
Tumor Associated Gene database

U

UCSC Genome Browser
UCSD-Nature Signaling Gateway Molecule Pages*
Uniclust
UniProt

V

ValidatorDB
ValidNESs*
VDJdb
VectorBase
VEuPathDB
VIDA*
VIOLIN
VIPERdb
ViPR
ViruSurf
Voronoia*
Voronoia4RNA*

W

WDSPdb*
WholeCellKB - Model Organism Databases for Comprehensive Whole-Cell Models
WITHDRAWN*
Wnt Database

Y

Yeast Resource Center

*Databases that have ceased operations as of August 15, 2022.
